# Molecular detection and genetic characterization of porcine circovirus 4 (PCV4) in Thailand during 2019–2020

**DOI:** 10.1038/s41598-023-32382-1

**Published:** 2023-03-30

**Authors:** Chaitawat Sirisereewan, Thanh Che Nguyen, Chutchai Piewbang, Suphattra Jittimanee, Roongtham Kedkovid, Roongroje Thanawongnuwech

**Affiliations:** 1 Department of Veterinary Pathology, Faculty of Veterinary Science, Chulalongkorn University, Bangkok, 10330 Thailand; 2 The International Graduate Program of Veterinary Science and Technology, Faculty of Veterinary Science, Chulalongkorn University, Bangkok, Thailand; 3 Research Group for Emerging and Re-emerging Infectious Diseases in Animals and Zoonotic Diseases, Faculty of Veterinary Medicine, Khon Kaen University, Khon Kaen, 40002 Thailand; 4 Division of Pathobiology, Faculty of Veterinary Medicine, Khon Kaen University, Khon Kaen, 40002 Thailand; 5 Department of Veterinary Medicine, Faculty of Veterinary Science, Chulalongkorn University, Bangkok, 10330 Thailand; 6 Center of Excellence in Swine Reproduction, Chulalongkorn University, Bangkok, Thailand; 7 Center of Excellence for Emerging and Re-emerging Infectious Diseases in Animals and One Health Research Cluster, Faculty of Veterinary Science, Chulalongkorn University, Bangkok, Thailand

**Keywords:** Genetics, Microbiology, Molecular biology, Diseases

## Abstract

Porcine circovirus 4 (PCV4) is considered a novel PCV, firstly found in China in 2019 and later discovered in Korea. This present study investigated the prevalence and genetic characteristics of PCV4 from high pig-density areas in Thailand during 2019–2020. From 734 samples, three samples (0.4%) from aborted fetuses and porcine respiratory disease complex (PRDC) cases were found positive for PCV4, two of the PCV4-positive samples were coinfected with both PCV2 and PRRSV, and the other PCV4-positive sample was found coinfected with PCV2. In situ hybridization (ISH) revealed the presence of PCV4 in the bronchial epithelial cells and in lymphocytes and histiocyte-like cells in the lymphoid follicles of the PRDC-affected pig. The complete Thai PCV4 genome had over 98% nucleotide identity with other PCV4 strains and was closely related to the Korean and Chinese PCV4b strains. Importantly, the amino acid residue at position 212 of the Cap gene is recommended for differentiating PCV4a (^212^L) from PCV4b (^212^M) based on currently available PCV4 genome sequences. These findings provide important clues for the pathogenesis, epidemiology, and genetic characteristics of PCV4 in Thailand.

## Introduction

To date, four species of porcine circoviruses (PCVs) have been identified, including PCV1, PCV2, PCV3, and PCV4^1^. PCV1 is nonpathogenic in pigs. PCV2 causes various symptoms collectively called porcine circovirus-associated diseases (PCVADs) or porcine circovirus diseases (PCVDs)^1–3^. PCV3 has been found in pigs with multiple clinical signs; however, the pathogenesis is still unknown^1,4,5^. Recently, a novel PCV4 has been identified in China and Korea both in clinically healthy and infected pigs with several clinical presentations, including respiratory and enteric signs and skin lesions suggestive of porcine dermatitis and nephropathy syndrome (PDNS)^6–11^. Therefore, despite having limited information on PCV4, the virus should not be overlooked. Furthermore, due to the lesson learned from past experiences with other swine viruses in Asia^12^, many emerging viruses have spread among countries due to the consequences of globalization through international trade and travel, both officially and unofficially. Therefore, the recent findings of PCV4 in China and Korea would raise awareness of the virus to the Asian swine practitioners to investigate this novel pathogen in their areas as part of the focus area “prevent and detect” to understand the disease distribution and its impact. In this study, we investigated from the samples obtained during 2019–2020 for the presence of PCV4 in Thailand and its genetic characterization.

## Results

### Prevalence and geographical distribution of PCV4 during 2019–2020

**Figure 1:**
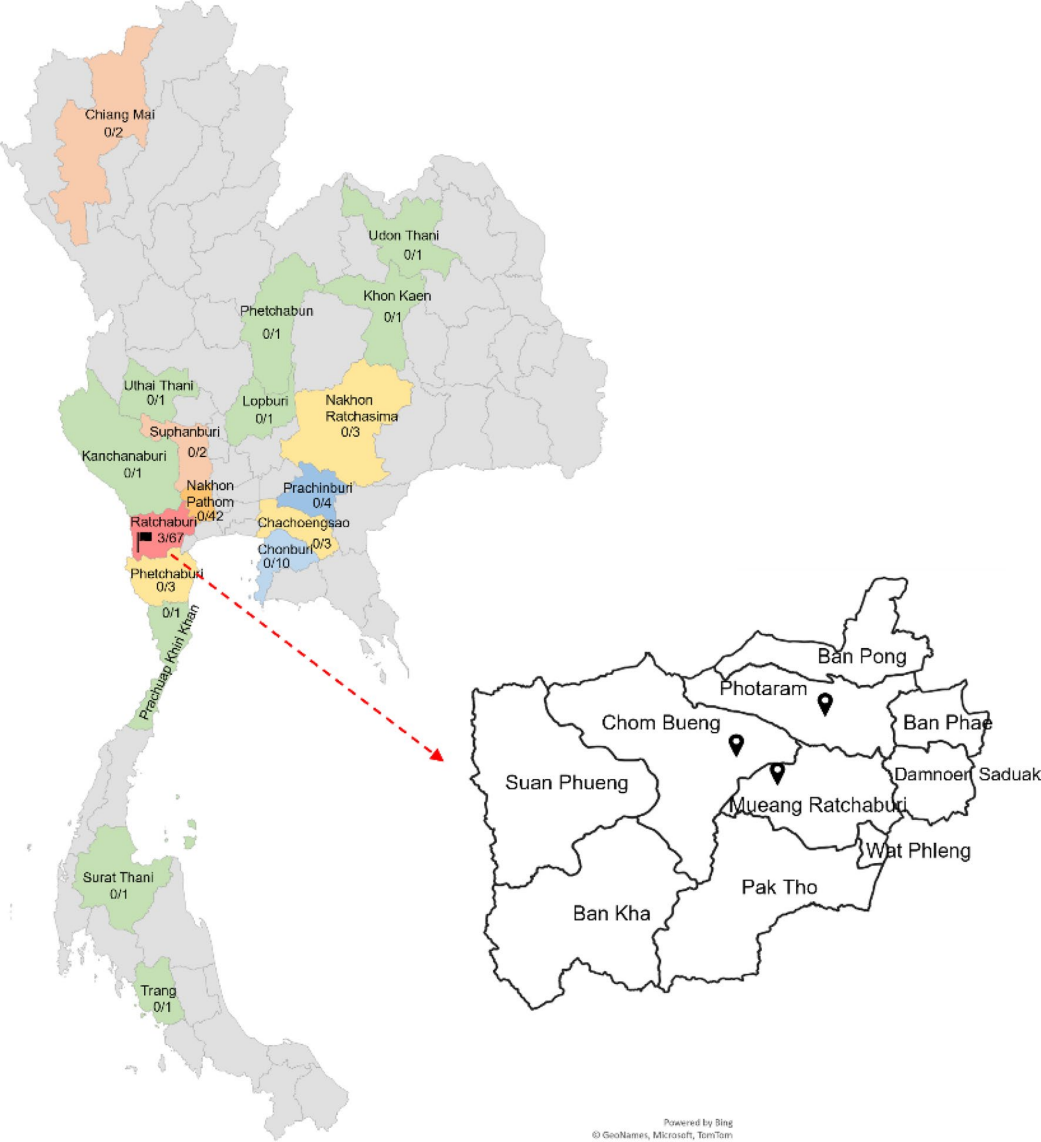
Geographic distribution of PCV4-tested swine farms (n = 145) in Thailand. The black flag indicates the Ratchaburi province where the positive samples were collected for complete genome sequencing. The locations of the 3 districts found PCV4-positive farms are indicated in the black pin.

To investigate the existence of PCV4, several types of swine samples (n = 734) from different geographical regions mainly located in the high pig density areas of Thailand were used. At sample levels, the overall positive rates of PCV4 were 0.4% (3/734). Notably, three PCV4-positive samples from clinically infected pigs with abortion and respiratory signs were found coinfected with PCV2 (3/3) and PRRSV (2/3) (Table 1). Among the 145 pig farms in these 18 provinces, 2.07% (3/145) were PCV4-positive. The geographic distribution of PCV4-positive farms was shown in Fig. 1.

**Table 1:** PCV4-positive samples found in Thailand during 2019–2020.

Collection year	Strain	Age of pigs	Sample	Location	Farm	History	Coinfection			
							PCV2	PCV3	PCV4	PRRSV
Jan-2019	19RBR246	Fetus	Pooled organs	Ratchaburi	Farm A	Abortion	Yes	–	Yes	–
Jan-2019	19RBR247	Grower-finishing	Pooled organs	Ratchaburi	Farm B	Respiratory signs, sudden death	Yes	–	Yes	Yes
Feb-2019	19RBR255	Nursery	Pooled organs	Ratchaburi	Farm C	Respiratory signs	Yes	–	Yes	Yes

### Genetic characteristics of PCV4

**Figure 2:**
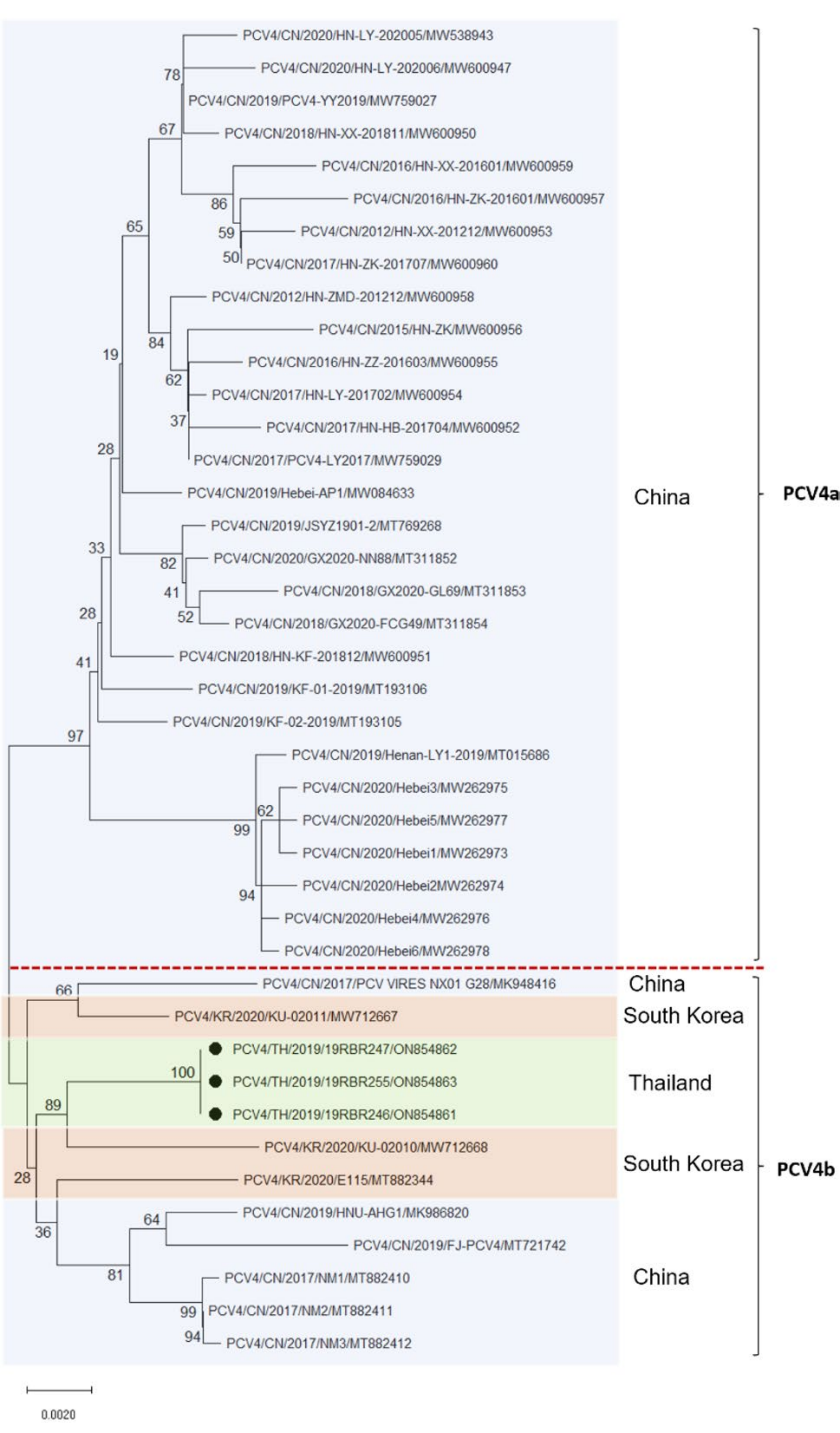
Phylogenetic tree based on the complete genome sequences of 3 Thai PCV4 strains and other reference strains. The Thai PCV4 sequences obtained in this study were marked with solid black circles. The colored background represented the country of origin of the PCV4 viruses. The phylogenetic tree was constructed using the neighboring-joining method with a *p*-distance model and bootstrapping at 1000 replicates.

In this study, all PCV4-positive samples were selected and sequenced for genetic characterization and phylogenetic analysis. The results showed that Thai PCV4 strains were 1770 nt in length, with 891 nt of ORF1 (Rep) and 687 nt of ORF2 (Cap). The complete genome sequences of the Thai PCV4 strains were aligned against those of reference viruses from China and Korea (Table 2). The results showed that the complete genome of the 3 Thai PCV4 strains shared 100% nucleotide identity to each other and shared 98.3–99.0% nucleotide identity with other reference PCV4 strains (Table 2). An amino acid sequence analysis indicated that the Rep and Cap genes of the Thai PCV4 strains shared similarity of 98.6%-100% and 97.8%-99.1%, respectively, when compared to each other and the reference strains. The Thai PCV4 strains showed the highest similarity to PCV4 NM2 from China with 99% nucleotide identity (complete genome), 100% Rep amino acid identity, and 98.6% Cap amino acid identity. Based on the phylogenetic analysis of complete genome nucleotide sequences of 41 PCV4 strains, the viruses have undergone evolution, resulting in two main distinct branches (PCV4a and PCV4b) (Fig. 2). The results showed that the Thai PCV4 strains belonged to PCV4b and clustered in the same branch with PCV4/KU-02010 strain found in Korea (Fig. 2). In the present study, PCV4 strains identified in China belong to both PCV4a and PCV4b genotypes. Interestingly, in the Korean and Thai swine farms, only PCV4b strains were detected. Moreover, the phylogenetic trees based on Cap and Rep genes were constructed for assessing genetic relationships (Supplementary Figure 1). The findings demonstrated that different genomic regions of PCV4 yielded similar outcomes.

**Table 2:** Percentage of nucleotide and amino acid identity (%) shared between Thai PCV4 strains and other reference strains.

Strain	Year	Accession no	Country	% Nucleotide identity (%amino acid identity)		
				Complete genome	Cap gene	Rep gene
19RBR246	2019	ON854861	Thailand	100	100 (100)	100 (100)
19RBR247	2019	ON854862	Thailand	100	100 (100)	100 (100)
19RBR255	2019	ON854863	Thailand	100	100 (100)	100 (100)
HNU-AHG1-2019	2019	MK986820	China	98.8	98.9 (98.6)	98.6 (99.6)
KU-02010	2020	MW712668	Korea	98.9	98.6 (98.2)	99.1 (100)
KU-02011	2020	MW712667	Korea	98.9	98.6 (98.6)	99.2 (100)
NM1	2017	MT882410	China	99	98.8 (98.6)	99.1 (100)
NM2	2017	MT882411	China	99	98.8 (98.6)	99.2 (100)
NM3	2017	MT882412	China	99	98.8 (98.6)	99.1 (99.6)
E115	2020	MT882344	Korea	98.8	98.9 (99.1)	98.8 (100)
PCV_VIRES_NX01_G28	2017	MK948416	China	98.7	98.3 (98.6)	99.3 (99.6)
FJ-PCV4	2019	MT721742	China	98.4	98.2 (97.8)	98.4 (98.6)
Hebei-AP1	2019	MW084633	China	98.9	98.8 (99.1)	99.2 (100)
Henan-LY1-2019	2019	MT015686	China	98.5	98.1 (98.6)	98.9 (99.6)
JSYZ1901-2	2019	MT769268	China	98.9	98.6 (99.1)	99.1 (99.6)
KF-01-2019	2019	MT193106	China	98.8	98.6 (99.1)	98.8 (99.3)
KF-02-2019	2019	MT193105	China	98.8	98.8 (99.1)	99.1 (100)
GX2020/NN88	2020	MT311852	China	98.7	98.6 (99.1)	98.9 (99.3)
GX2020/GL69	2018	MT311853	China	98.6	98.5 (98.2)	98.8 (99.3)
GX2020/FCG49	2018	MT311854	China	98.8	98.8 (99.1)	98.9 (99.6)
HN-LY-202005	2020	MW538943	China	98.6	98.5 (98.6)	98.9 (99.6)
HN-LY-202006	2020	MW600947	China	98.5	98.6 (98.6)	98.6 (98.9)
HN-XX-201811	2018	MW600950	China	98.8	98.8 (99.1)	98.9 (99.3)
HN-KF-201812	2018	MW600951	China	98.8	98.6 (98.6)	99.1 (100)
HN-HB-201704	2017	MW600952	China	98.5	98.3 (97.8)	98.9 (99.6)
HN-XX-201212	2012	MW600953	China	98.4	98.6 (99.1)	98.7 (100)
HN-LY-201702	2017	MW600954	China	98.7	98.6 (98.6)	98.9 (100)
HN-ZZ-201603	2016	MW600955	China	98.6	98.3 (98.2)	99.1 (100)
HN-ZK	2015	MW600956	China	98.4	98.3 (97.8)	98.6 (98.9)
HN-ZK-201601	2016	MW600957	China	98.3	98.3 (98.2)	98.5 (98.6)
HN-ZMD-201212	2012	MW600958	China	98.7	98.6 (99.1)	98.9 (99.6)
HN-XX-201601	2016	MW600959	China	98.4	98.3 (98.6)	98.8 (98.9)
HN-ZK-201707	2017	MW600960	China	98.6	98.8 (99.1)	98.8 (99.3)
LY2017	2017	MW759029	China	98.8	98.6 (98.6)	99.1 (100)
YY2019	2019	MW759027	China	98.8	98.8 (99.1)	98.9 (99.6)
Hebei1	2020	MW262973	China	98.4	97.8 (98.2)	98.9 (99.6)
Hebei2	2020	MW262974	China	98.4	97.6 (97.8)	98.9 (99.6)
Hebei3	2020	MW262975	China	98.4	97.9 (98.6)	98.8 (99.6)
Hebei4	2020	MW262976	China	98.4	97.9 (98.6)	98.8 (99.3)
Hebei5	2020	MW262977	China	98.4	97.9 (98.6)	98.8 (99.3)
Hebei6	2020	MW262978	China	98.4	97.8 (98.6)	98.9 (99.6)

### Nucleotide sequence comparison and amino acid sequence analysis of PCV4

**Figure 3:**
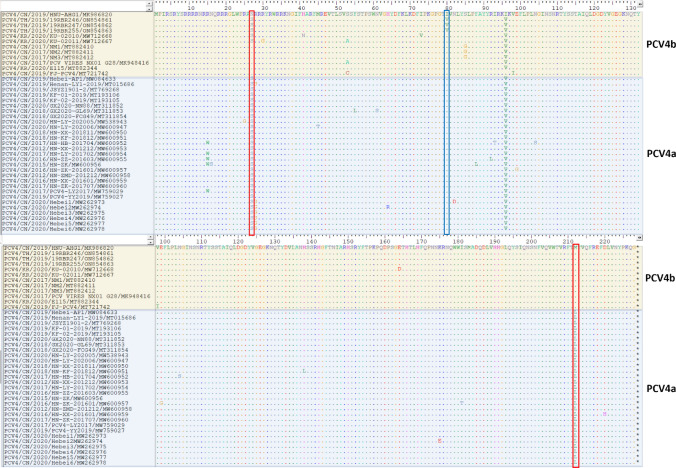
Comparison of Cap amino acid sequences of PCV4 isolates in this study. Dots are used to denote the residues that are consistent with HNU-AHG1 (MK986820). The red boxes indicate the amino acid at positions 27 and 212, previously proposed for differentiation of PCV4a and PCV4b. Mutations at residues position 80 of Thai PCV4 strains are shown with the green box.

An analysis of nucleotide and amino acid sequences of the Cap and Rep genes against reference PCV4 strains demonstrated that Thai PCV4 genomes displayed nucleotide identities of 97.6%-98.9% amino acid and 98.4%-100% amino acid in Cap gene and Rep genes, respectively compared to those of reference viruses. The Thai PCV4 strains shared amino acid identities of 97.8%-99.1% and 98.6%-100% in Cap gene and Rep gene compared to those of reference viruses. There were no amino acid deletions or insertions. Compared with prototype PCV4 strain HNU-AHG1-2019 (accession no. MK986820), Thai PCV4 genomes had 20 nucleotide substitutions (Table 3). For deduced amino acid analysis, one amino acid substitution (Q155K) was seen in Rep gene, while 3 amino acid substitutions (N27S, I80V, and I96V) were found in Cap gene (Fig. 3). Among them, there was one unique amino acid substitution (I80V) in the Thai PCV4 strains of Cap gene compared with other PCV4 strains. Compared with the representative strains (Fig. 3), the Cap gene of PCV4a contains specific amino acid patterns of 27S and 212L, while certain PCV4b strains have unique amino acid patterns of 27N and 212M. Interestingly, the sequences from the present study revealed that the Thai PCV4 strains were grouped in PCV4b, even though they showed amino acid 27S in the Cap gene, and possessed the same amino acid at position 212M, similar to the two PCV4 strains (KU-02010 and KU-02011) from Korea. The findings suggest that amino acid variation at position 212 of Cap gene could be used for differentiating PCV4a (^212^L) from PCV4b (^212^M) (Fig. 3).

**Table 3:** Nucleotide sequence comparison between Thai PCV4 strain (19RBR247) and the prototype PCV4 strain HNU-AHG1-2019.

Genome position	PCV4 HNU-AHG1-2019	PCV4 19RBR247
36	A	G
122	T	C
206	C	T
287	C	T
432	C	T
537	C	A
557	T	C
599	T	C
608	A	C
726	C	T
866	C	T
896	C	T
920	G	A
1251	T	G
1260	G	A
1448	T	C
1496	T	C
1527	C	T
1654	T	C
1680	G	T

### Detection of PCV4 by in situ hybridization

**Figure 4:**
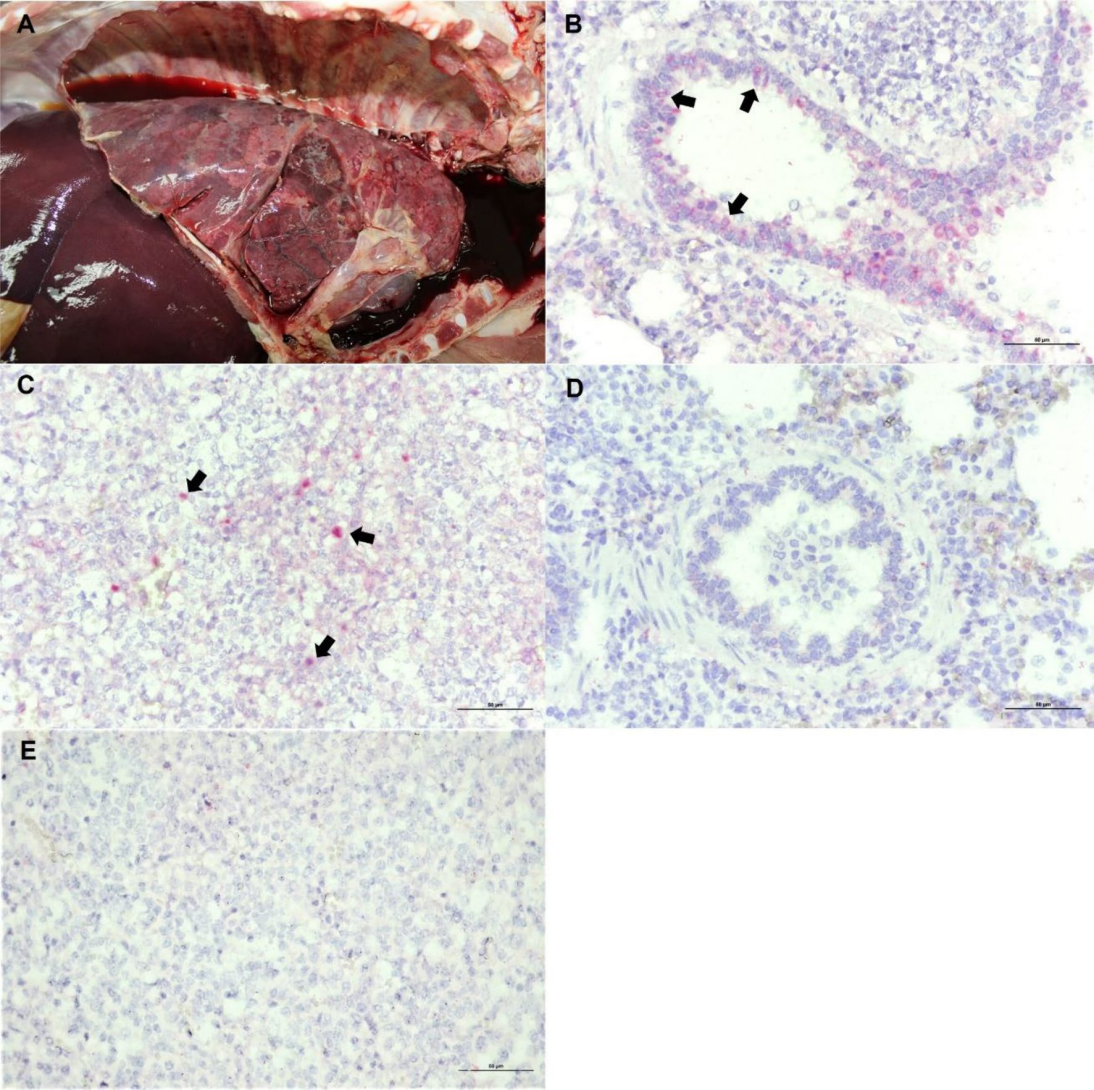
Gross and microscopic lesions and in situ hybridization (ISH) using PCV4-specific probe targeting Cap gene of PCV4. Grossly, lung failed to collapse with dark red to purple consolidation and diffuse fibrinous attachment in the pleura (**A**). Lung: PCV4-positive cells were characterized by pink to brilliant red in the cytoplasm of bronchial epithelial cells (arrows) (**B**). Tracheobronchial lymph node: positive signals were observed in lymphocytes and histiocyte-like cells in the lymphoid follicle (arrows) (**C**). No hybridization signals were seen in the internal negative control of lung and tracheobronchial lymph node (**D**,**E**).

The PCV4-positive pig (19RBR247) with respiratory illness was submitted for necropsy. Prominently, lungs with enlarged tracheobronchial lymph nodes were diffusely mottled and failed to collapse with dark red to purple consolidation and diffuse fibrinous attachment in the pleura (Fig. 4A). Microscopically, lungs revealed moderate to severe diffuse pulmonary and interlobular edema with mild multifocal hyperplasia of bronchus-associated lymphoid tissue, and moderate to severe diffuse broncho-interstitial pneumonia. Tracheobronchial lymph nodes showed moderate multifocal lymphoid depletion. To investigate the tissue localization of PCV4, lung and tracheobronchial lymph node were tested using in situ hybridization. The results showed that PCV4-ISH-positive signals were mainly observed in the cytoplasm of bronchial epithelium (Fig. 4B). Moreover, a few positive signals were detected in the lymphocytes and histiocyte-like cells of the tracheobronchial lymph node (Fig. 4C). No hybridization signals were seen in internal negative control of lung (Fig. 4D) and tracheobronchial lymph node tissue sections (Fig. 4E).

## Discussion

PCV2 and PCV3 are at least the two porcine circoviruses causing major threats to the global swine industry^1–3^. The finding of PCV4 should not be neglected since Asia is the major world hub of pig production, and the virus itself may originate here and spread to other regions or vice versa. To date, PCV4 was discovered only in the Asian continent, China, Korea, and recently, Thailand, but not yet in others^6–11,13,14^.

In the present study, the results showed that only 3 of the 734 samples were tested positive for PCV4, with a positive rate of 0.4% (3/734) and 2.07% (3/145) of farm levels. Interestingly, extremely low prevalence of PCV4 was demonstrated in Thailand compared to previous reports^6,7,9–11^ suggesting low transmission and infection rate within the pig herds. However, nearly 80% of the swine farms tested were mainly located in the high pig density areas of Thailand, which might not reflect the whole picture on PCV4 infection status of the country. Thus, in the future, large-scale field sampling of all regions could provide more information about PCV4 epidemiology in Thailand. Therefore, the threat of PCV4 to the Thai swine industry is skeptical and yet to be elucidated.

Recently, pigs inoculated with rescued PCV4 alone showed histopathological changes in several organs, but no obvious clinical signs found^15^. In the present study, we found that three PCV4-positive samples from clinically infected pigs were found coinfected with PCV2 (3/3) and PRRSV (2/3). These results apparently suggest that absence of other factors, such as coinfections, PCV4 infection might remain asymptomatic. However, the synergistic effect of PCV4 infection might exacerbate the disease severity and be associated with PRDC in clinically infected pigs as found in the present study. Therefore, co-infections and some unknown factors, yet to be elucidated, might involve PCV4 pathogenesis and its clinical outcomes.

According to in situ hybridization results, the virus presence was found in the bronchiolar epithelial cells and in lymphocytes and histiocyte-like cells in the lymphoid follicles consistently with the characteristics of PCV2 infection^16^. Whether PCV4 infection in bronchiolar epithelial cells and tracheobronchial lymph node induces the observed broncho-interstitial pneumonia and the lymphoid depletion should be further studied. The contribution of PCV4 to the pathogenesis of PRDC and immune modulation should be of interest. These findings also suggest that a marked tropism of PCV4 for bronchial epithelial cells may impair the epithelial barrier function, thus predisposing the infected pigs to secondary infections and PRDC. The case history of the PCV4 positive cases found that the PCV4-infected pigs were found coinfected with PCV2, PRRSV, and/or *Streptococcus* spp. Additionally, bronchial epithelial infection might contribute to viral shedding dynamics in the nasal secretion. Notably, it has been reported that the highest positive rates of PCV4 were detected in nasal swabs followed by serum samples^6^. Therefore, nasal swabs might be a better target specimen for PCV4 detection and surveillance. Further investigation is needed.

For genetic analysis, Thai PCV4 strains shared up to 98% nucleotide identity with other reference PCV4 strains. The high genetic stability of PCV4 is consistent with the previous reports^7,11^. It is noted that the Thai PCV4 strains were closely related to the Chinese PCV4 sub-cluster. Although the virus had high genetic stability, some genetic variations could be observed among pig populations from different countries that probably specific to the geographic origin^17^. Additionally, the Thai PCV4 strains were highly related to each other, possibly, due to a single point introduction with low infection rate or restricted gene flow in the neighboring districts of Western Thailand, suggesting that the novel virus might be confined to these areas affecting the prevalence and genetic diversity.

Additionally, there are currently no fully established guidelines for classifying PCV4 genotypes, and the temporary proposals made so far have not been consistent. This is in contrast with PCV2 genotyping that a unified classification scheme based on Cap gene was proposed^18^. For PCV4 genotyping, there have been several studies proposing different criteria and markers to differentiate PCV4 genotypes, such as 2-group classification (PCV4a and PCV4b) and 3-group classification (PCV4a, PCV4b, and PCV4c)^10,19–21^. Among these studies, the residue at 27 and 212 of capsid gene were also proposed to be used in distinguishing PCV4a (27S and 212L) from PCV4b (27N and 212M)^19,20^. Recently, PCV4c was proposed with specific amino acid pattern 27N, 28R, and 212M. However, in some genotyping criteria, PCV4c might be classified into PCV4b^10,20^. Therefore, the use of differing nomenclatures can create ambiguity and misinterpretation of results. Further investigation is required to establish the standardized criteria for genotyping PCV4, given the current paucity of sequence information. As mentioned above, previous reports showed that the amino acid at the position 27 of the Cap gene could be used as a marker to distinguish between PCV4a (27S) and PCV4b (27N)^19,20^. However, the results in this study showed that the amino acid residue at position 27 cannot be used to differentiate between PCV4a and PCV4b since the Thai PCV4b strains and the Korean PCV4b strains (KU-02010 and KU-02011) showed N27S (Fig. 3). The novel findings of this study suggest that amino acid variation at position 212 could be used for differentiating PCV4a (212L) from PCV4b (212M) for 2-group classification. Moreover, one unique amino acid substitution (I80V) was also found in the Thai PCV4 strains in Cap gene. The amino acid substitution occurred at residue 72–88 in B-cell epitopes on PCV4 capsid gene^20^. It may alter antigenic properties of the viruses and their immunogenic modifications caused by genetic mutation. However, the effects of genetic mutation on pathogenicity need further investigation. In terms of the genetic variation of PCV4 and its genotypes, a study conducted in various regions in China found that PCV4a was the most common genotype followed by PCV4b^10,11,19–21^. However, in pig populations from Korea and Thailand, only the PCV4b genotype was detected^8^. It is plausible to consider that PCV4 strains isolated from various geographic regions or pig populations may potentially demonstrate genetic variations, which may imply the existence of geographical or host specificity in the virus.

To date, PCV4 was discovered only in the Asian continent, China, Korea, and recently Thailand^6–11,13,14^. This study described the presence of PCV4 in Thailand from the retrospective samples and firstly demonstrated the viral tropism in the bronchial epithelial cells. The three Thai PCV4 strains demonstrated their genetic similarity with the Korean and Chinese PCV4b strains. However, the prevalence of PCV4 in Thailand is extremely low, and clinical involvement of PCV4 remains unclear. Apparently, it should be noted that the amino acid residue at position 212 of the Cap gene should be used for differentiating PCV4a and PCV4b. Further studies are needed to determine the role of PCV4 infection related to clinical signs and its impact on the Thai swine farms. The classification of PCV4 genotypes requires further investigation and clarification due to the limited available PCV4 sequences.

## Materials and methods

### Sample collection and viral DNA extraction

Seven hundred thirty-four samples from 145 swine farms submitted for diagnostic purposes at the Chulalongkorn University, Veterinary Diagnostic Laboratory (CU-VDL), and Diagnostic Laboratory of Large Animal Hospital and Students Training Center during January 2019- December 2020 were used for this study. The samples consisted of serum (n = 426), tissue (n = 188), fetus (n = 75), semen (n = 25), feces (n = 16), colostrum (n = 2), and oral fluid (n = 2) that were mainly collected from the high pig density areas in the Western, Central, and Eastern parts of Thailand.

Total viral DNA was extracted using IndiMag Pathogen kit of viral RNA/DNA (Indical Bioscience, Germany) following the manufacturer's instruction. The extracted DNA was stored at -80 °C until used.

### Molecular detection of PCV4

TaqMan® real-time PCR was performed to detect PCV4 targeting the replicase gene (rep) of PCV4 using two newly designed primer pairs and probe (Supplementary Table S1). Briefly, PCR reactions were performed in a total 20 µl reaction containing 0.4 µM of forward and reverse primers, 0.2 µM of probes, 10 µl of Luna® Universal Probe qPCR master mix (NEB, MA, USA), and 3 µl of extracted DNA. The PCR condition consisted of initial denaturation at 95 °C for 60 s followed by 45 cycles of 95 °C for 15 s and 60 °C for 30 s using Quantstudio5 real-time system (Applied Biosystems, USA). Positive control plasmid was synthesized by inserting a full length of PCV4 rep gene (891 bp) into the pUC18 vector by GenScript Company (Nanjing, China). Detection limit of the PCV4 TaqMan® real-time PCR was 200 copies/µl of standard plasmid DNA.

### Complete genome amplification and sequencing

The complete genomes of PCV4-positive samples were amplified using two primer sets (Supplementary Table S1). The PCR reactions were performed in 25 µl reaction mixtures containing 3 µl of extracted DNA, 0.5 µM of forward and reverse primers, and 12.5 µl of Q5^®^ High-Fidelity 2 × master mix. The PCR thermal profile involved an initial denaturation of 98 °C for 30 s followed by 35 cycles of 98 °C for 10 s, 72 °C for 30 s, 72 °C for 50 s, and final extension at 72 °C for 2 min. The PCR products were purified using Nucleospin™ Gel and PCR clean-up (MACHEREY–NAGEL, Germany) and submitted for sequencing by a barcode-tagged sequencing platform (Celemic, Seoul, Korea). The obtained nucleotide sequences were further analyzed and assembled with SeqMan, and Editseq software v.5.03 (DNASTAR Inc., Madison, Wisconsin, USA), then deposited in GenBank under accession no. ON854861-ON854863.

### Phylogenetic analysis

For pairwise comparison and genetic characterization of PCV4, the complete nucleotide sequences were aligned using the Clustal W algorithm of BioEdit 7.2.5 (https://bioedit.software.informer.com/) with the reference PCV4 strains from the GenBank database. Phylogenetic trees were reconstructed with MEGA version 10.2.6 using the neighbor-joining algorithm (NJ) with 1000 bootstrap replicates^22^.

Currently, guidelines for identifying PCV4 genotypes are not yet fully established, but they have been temporarily proposed and were not consistent. Generally, PCV4 strains have been classified into two main genotypes, PCV4a and PCV4b^10,19,20^. However, a single study has proposed that PCV4 might be classified into three genotypes, including PCV4c^21^. To simplify the analysis, in this present study, PCV4 strains were classified into two main genotypes, PCV4a and PCV4b. The phylogenetic analyses were carried out using the complete genome, Rep gene, and Cap gene, following the approach previously described^10^.

### Detection of PCV4 in tissues using in situ hybridization

Of the three PCV4-positive samples, the formalin-fixed paraffin-embedded (FFPE) tissue samples were available for only one case, 19RBR247, and thus used for virus localization analysis. The FFPE tissues were stained with hematoxylin and eosin (HE) for histopathology study. To identify PCV4 tissue localization, in situ hybridization (ISH) was performed according to a previously described protocol with some modifications^23^. Briefly, PCV4-specific probe targeting 110 bp of the Cap gene of PCV4 was constructed using a PCR DIG Probe Synthesis Kit (Roche Diagnostics, Basel, Switzerland) following the manufacturer’s instructions. The sections were incubated overnight at 40 °C in a moist chamber with PCV4-specific probe. The PCV4-specific DIG probe was detected by using 1:200 anti-DIG AP Fab fragments (Roche Diagnostics, Basel, Switzerland). Hybridization signals were detected as pink to brilliant dark red colorimetric staining of Permanent Red (LPR) (Dako, Glostrup, Denmark) with 50% hematoxylin counterstaining. Slides incubated without the DIG probe were used as a negative control.

## Supplementary Information

Supplementary Information.

## Data Availability

The datasets generated and analysed during the current study are available in the NCBI genomes repository under the accession numbers ON854861-ON854863.
