# Supplementary material for: Molecular detection and genetic characterization of porcine circovirus 4 (PCV4) in Thailand during 2019–2020
Source: Sci Rep. 2023 Mar 30;13:5168. doi: 10.1038/s41598-023-32382-1 (PMC10063680; doi:10.1038/s41598-023-32382-1)
Supplement: Supplementary file 1 — Supplementary Information. [file 41598_2023_32382_MOESM1_ESM.docx]

**Molecular detection and genetic characterization of porcine circovirus 4 (PCV4) in Thailand during 2019-2020**

Chaitawat Sirisereewan^1^, Thanh Nguyen Che^2^, Chutchai Piewbang^1^, Suphattra Jittimanee^3,4^, Roongtham Kedkovid^5,6*^, Roongroje Thanawongnuwech^1,7*^

^1^Department of Veterinary Pathology, Faculty of Veterinary Science, Chulalongkorn University, Bangkok, Thailand

^2^The International Graduate Program of Veterinary Science and Technology, Faculty of Veterinary Science, Chulalongkorn University, Bangkok, Thailand

^3^Research Group for Emerging and Re-emerging Infectious Diseases in Animals and Zoonotic Diseases, Faculty of Veterinary Medicine, Khon Kaen University, Khon Kaen 40002, Thailand.

^4^Division of Pathobiology, Faculty of Veterinary Medicine, Khon Kaen University, Khon Kaen 40002, Thailand.

^5^Department of Veterinary Medicine, Faculty of Veterinary Science, Chulalongkorn University, Bangkok, Thailand

^6^Center of Excellence in Swine Reproduction, Chulalongkorn University, Bangkok, Thailand

^7^Center of Excellence for Emerging and Re-emerging Infectious Diseases in Animals and One Health Research Cluster, Faculty of Veterinary Science, Chulalongkorn University, Bangkok, Thailand

**
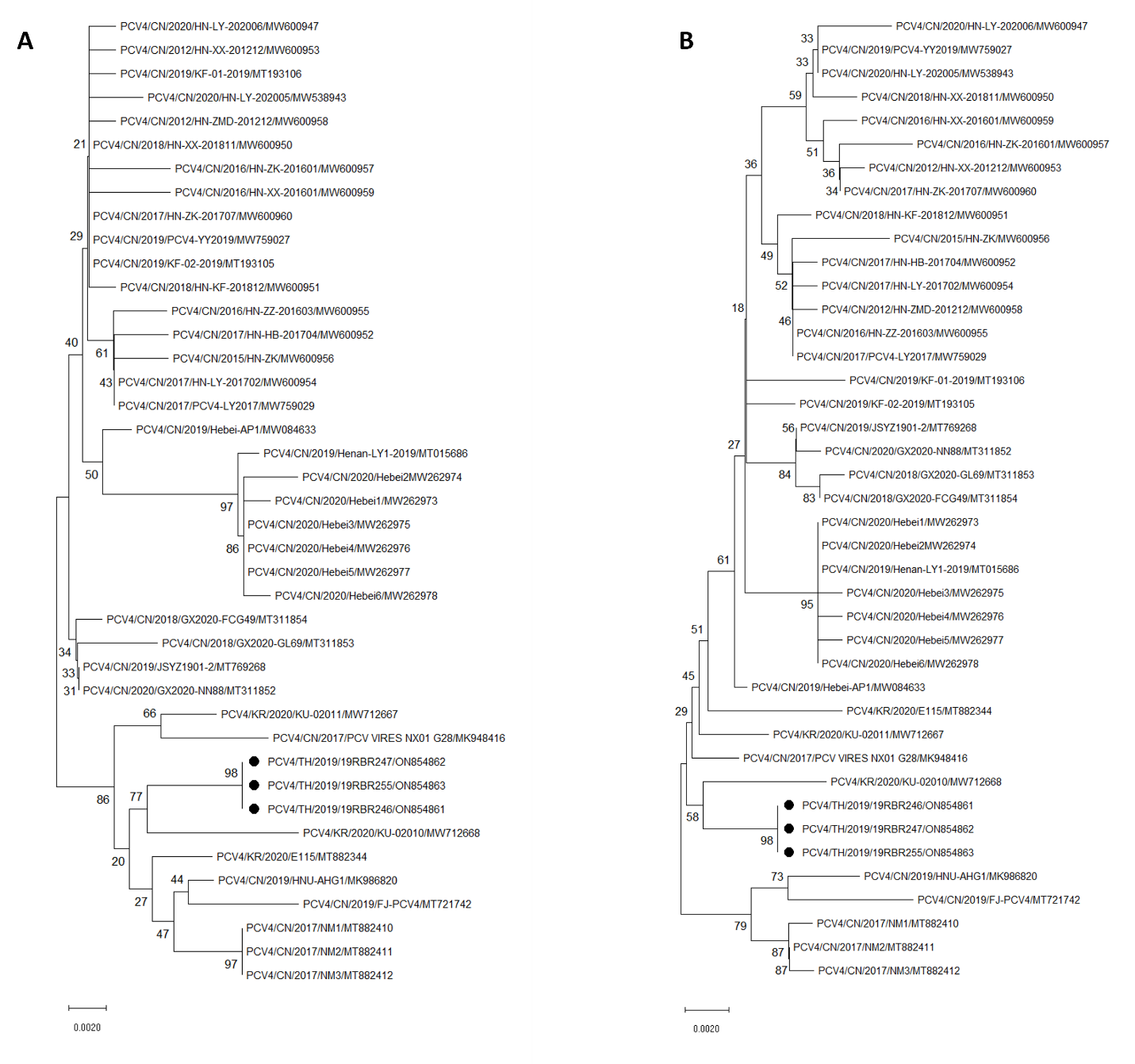
**

**Supplementary figure 1.** Phylogenetic tree based on full length of *Cap* gene (A) and *Rep* gene (B) using the neighboring-joining method with a *p*-distance model and bootstrapping at 1000 replicates. The Thai PCV4 sequences obtained in this study were marked with solid black circles.

**Supplementary table S1.** List of primers sequences used for detection and sequencing Porcine circovirus 4 (PCV4) in this study.

| Primer | Sequences (5’-3’) | Reference |
| --- | --- | --- |
| PCV4-rF | AAAGCGCAGCGACCTTAAAG | For detection, this study. |
| PCV4-rR | CACGGGCCACTTCACTCATT |  |
| PCV4-Probe | ROX-CTGTGGCCGCCCTGAATGCC-BHQ2 |  |
| PCV4-277F | GTGGCCTCCGGGACTACGTCAT | For genome sequencing^1^. |
| PCV4-170R | GCACTGGGCTCTCCTACTTCCAG |  |
| PCV4-88F | GCCACCCCGTGAAGAGATATT | For genome sequencing, this study. |
| PCV4-586R | CCGGGGATACCCACGATGAC |  |

**Reference**

1 Zhang, H. H. *et al.* Novel circovirus species identified in farmed pigs designated as Porcine circovirus 4, Hunan province, China. *Transboundary and Emerging Diseases* **67**, 1057-1061, doi:<https://doi.org/10.1111/tbed.13446> (2020).
